# Function and Evolution of Two Forms of SecDF Homologs in *Streptomyces coelicolor*

**DOI:** 10.1371/journal.pone.0105237

**Published:** 2014-08-20

**Authors:** Zhan Zhou, Yudong Li, Ning Sun, Zhihao Sun, Longxian Lv, Yufeng Wang, Libing Shen, Yong-Quan Li

**Affiliations:** 1 Province Key Laboratory of Microbial Biochemistry and Metabolism Engineering, College of Life Sciences, Zhejiang University, Hangzhou, Zhejiang, China; 2 Department of Bioengineering, School of Food Sciences and Biotechnology, Zhejiang Gongshang University, Hangzhou, Zhejiang, China; 3 Department of Biology and South Texas Center for Emerging Infectious Diseases, University of Texas at San Antonio, San Antonio, Texas, United States of America; 4 State Key Laboratory of Genetic Engineering and Ministry of Education Key Laboratory of Contemporary Anthropology, School of Life Sciences, Fudan University, Shanghai, China; University Paris South, France

## Abstract

The general secretion (Sec) pathway plays a prominent role in bacterial protein export, and the accessory component SecDF has been shown to improve transportation efficiency. Inspection of *Streptomyces coelicolor* genome reveals the unexpected presence of two different forms of *secDF* homologous genes: one in fused form (*secDF*) and the other in separated form (*secD* and *secF*). However, the functional role of two SecDF homologs in *S. coelicolor* has not yet been determined. Transcriptional analysis of *secDF* homologs reveals that these genes are constitutively expressed. However, the transcript levels of *secD* and *secF* are much higher than that of *secDF* in *S. coelicolor*. Deletion of *secDF* or/and *secD/secF* in *S. coelicolor* did result in reduced secretion efficiency of Xylanase A and Amylase C, suggesting that they may have redundant functions for Sec-dependent translocation pathway. Moreover, our results also indicate that SecD/SecF plays a more prominent role than SecDF in protein translocation. Evolutionary analysis suggests that the fused and separated SecDF homologs in *Streptomyces* may have disparate evolutionary ancestries. SecD/SecF may be originated from vertical transmission of existing components from ancestor of *Streptomyces* species. However, SecDF may be derived from bacterial ancestors through horizontal gene transfer. Alternately, it is also plausible that SecDF may have arisen through additional gene duplication and fusion events. The acquisition of a second copy may confer a selective benefit to *Streptomyces* by enhancing protein transport capacity. Taken together, our results provide new insights into the potential biological function and evolutionary aspects of the prokaryotic SecDF complex.

## Introduction

Streptomycetes are soil-dwelling Gram-positive bacteria that have a large (6.5–11.9 Mb) linear chromosome with high GC content, containing a central region that is highly conserved throughout the genus, and terminal regions that are variable in composition and organization [1], [2]. *Streptomyces* species secreted large quantities of proteins via the general secretory (Sec) pathway and twin-arginine translocation (Tat) pathway to their living niches [3]–[6]. These systems could facilitate nutrient acquisition by secreting chitinases and cellulases to degrade insoluble nutrient sources, and regulate morphological development of *Streptomyces* by secreting peptidases and their associated inhibitors [5]. Moreover, *Streptomyces* has become an important production host for heterologous expression of recombinant proteins due to its excellent secretion capacity, which makes the downstream processing much simpler [7]–[10]. Therefore, understanding of the mechanism of its protein export systems is extremely important for the economical application of *Streptomyces*.

Protein transport across the bacterial membrane is mediated by different translocation systems, of which the general protein secretion (Sec) system plays a prominent role in protein export and membrane insertion [11]–[15]. The Sec translocation machinery is a protein complex comprised of SecYEG (SecY, SecE, SecG), the ATPase SecA, and the accessory factor SecDF (SecD and SecF) [14]–[16]. The roles of the Sec pathway components have been extensively studied, but the function of the SecDF complex in protein secretion is still poorly understood. The SecDF complex has been shown to be involved in the cycling of SecA and the release of the translocated protein from the translocation channel [17], [18]. Recently, the SecDF complex was proposed to be a membrane integrated chaperone that uses proton motive force (PMF) to complete protein translocation through the SecYEG channel via the control of SecA cycling [19].

The SecDF protein complex belongs to the resistance-nodulation-cell division (RND) family of multidrug export pumps, which is widely distributed and conserved in all three major kingdoms of life. However, the SecDF complex is only found in kingdoms Bacteria and Archaea [20]. The SecDF complex is comprised of two structurally related proteins, SecD and SecF, which have previously been shown to have arisen via an internal gene duplication event [21]. The *secD* and *secF* genes are adjacent in genomes and widely distributed in bacteria, such as *Escherichia coli* [22]. Interestingly, the fusion form of *secD* and *secF* genes, *secDF*, has also been observed in bacteria, such as *Bacillus subtilis* and *Staphylococcus aureus* [23], [24]. These studies showed, for example, that both SecDF in *B. subtilis* [23] and SecD/SecF in *E. coli* [25] are involved in catalyzing protein translocation. The existence of the two forms of *secDF* genes shows different evolutionary pathways of SecDF complex. However, the evolutionary processes still remain obscure.

Here, we report the presence of two different forms of *secDF* genes, one separated (*secD*/*secF*, hereafter *secD-F*) and one fused (*secDF*) in the genome of *S*. *coelicolor* [26]. To further understand the auxiliary functions of SecDF and SecD-F, these genes were deleted together or separately. Our results here show that the simultaneous depletion of these genes results in severe inhibition of Xylanase A (XlnA) and Amylase C (AmlC) secretion, suggesting that SecDF and SecD-F may have redundant functions in the Sec protein translocation pathway. Moreover, phylogenetic analysis supported the proposal that the fused and separated *secDF* homologs of *Streptomyces* may have different evolutionary ancestries.

## Materials and Methods

### Bacterial strains, plasmids and growth conditions

Bacterial strains and plasmids used in this study are listed in Table 1, and primers are listed in Table S1 in File S1. The wild type strain M145 of *S. coelicolor* A3(2) strain was used for construction of a series of mutant strains in this study (Table 1). *E. coli* TG1 was used for gene cloning and plasmid construction. The non-methylating *E. coli* ET12567 containing plasmid pUZ8002 was used for conjugation with *Streptomyces* for transduction of plasmids.

**Table 1:** Strains and plasmids used in this study.

Strains/Plasmids	Description	Reference or source
**Strains**		
*E. coli* TG1	Used for gene clone	[30]
*E. coli* ET12567/pUZ8002	Non-methylating *E. coli*, containing pUZ8002, for conjugation with *Streptomyces*	[30]
*S. coelicolor* M145	*Streptomyces coelicolor* A3(2), SCP1^−^, SCP2^−^	[26]
ZJUZ1	M145, *ΔsecD-F*::Null	This study
ZJUZ2	M145, *ΔsecDF:*:Null	This study
ZJUZ3	M145, *ΔsecD-F*::Null, *ΔsecDF*::Null	This study
ZJUZ23	M145+pZJ6	This study
ZJUZ24	ZJUZ1+pZJ6	This study
ZJUZ25	ZJUZ2+pZJ6	This study
ZJUZ26	ZJUZ3+pZJ6	This study
ZJUZ27	M145+pZJ7	This study
ZJUZ28	ZJUZ1+pZJ7	This study
ZJUZ29	ZJUZ2+pZJ7	This study
ZJUZ30	ZJUZ3+pZJ7	This study
ZJUZ39	M145+pIJ8630	Lab store
ZJUZ40	M145+pZJ9	This study
ZJUZ41	M145+pZJ10	This study
LM6	M145+pLM1	[32]
**Plasmids**		
pTA2	Used for TA clone, *Amp, LacZ*	TOYOBO
pKC1139	Used for gene knock-out, *aac3(IV)*	[29]
pZJ2	Expression vector, *rep* ^pIJ101^, *rep* ^pUC^, *oriT*, *tsr*, *aac3(IV)*	This study
pIJ8630	Promoter detecting vector, aac3(IV)	[31]
PLM1	pIJ8630-ermEp*	[32]
pZJ9	pIJ8630-*secDp* for promoter detection	This study
pZJ10	pIJ8630-*secDFp* for promoter detection	This study
pKC1139a	pKC1139-*secD* left arm-*secF* right arm for knock-out	This study
pKC1139b	pKC1139-*secDF* left arm-right arm for knock-out	This study
pZJ6	pZJ2-*xlnA* with own promoter	This study
pZJ7	pIJ8630-ermEp*-amlC	This study

ermEp*: the enhanced promoter region of the erythromycin resistance gene (*ermE*) of *Streptomyces erythraeus* [48].

*E. coli* strains were routinely cultured in Lennox broth (LB) liquid medium or on agar plates at 37°C. *S. coelicolor* strains were cultured at 30°C in liquid tryptic soy broth (TSB) or YEME medium for mycelium preparation in the primary metabolism, or in M14 medium [27] for the xylanase activity assay. Solid MM, SMMS and R2YE medium were used for cell differentiation, and MSF medium for spore preparation of *S. coelicolor* [28].

### Construction of *S. coelicolor* mutant strains

**Figure 1:**
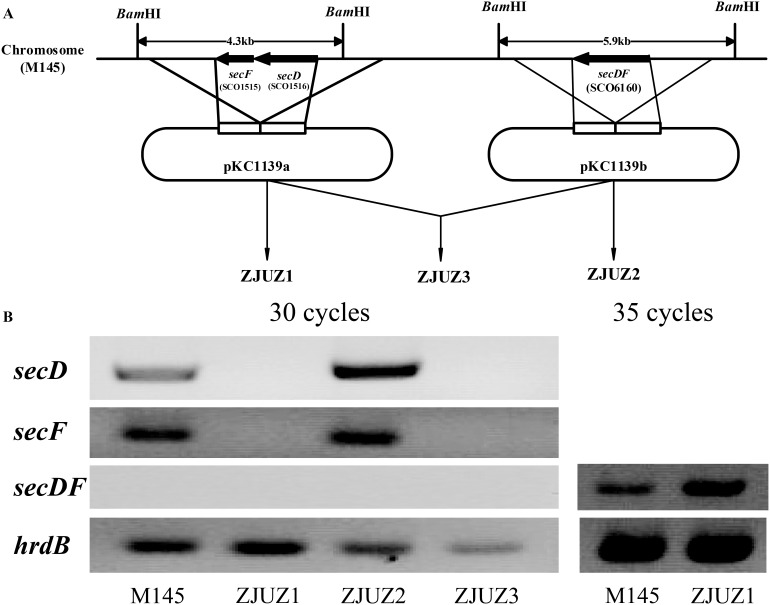
The gene knock out processes and gene expression profiles of *secDF* homologous genes in *S. coelicolor*. (A) Schematic representation of the in frame deletion of *secD-F* and *secDF* by double-crossover homologous recombination in *S. coelicolor* M145. Plasmids pKC1139a and pKC1139b were used to knock out *secD-F* (ZJUZ1) and *secDF* (ZJUZ2), respectively. Both pKC1139a and pKC1139b were used to create the ZJUZ3, which lacks both *secD-F* and *secDF* genes. (B) Gene expression analysis of *secD*, *secF* and *secDF* in *S. coelicolor* wild type and mutant strains by RT-PCR with 30 and 35 cycles. Gene hrdB was used as a reference gene.

The *secD*, *secF* and *secDF* genes were disrupted by in frame deletion via double-crossover homologous recombination [28] with the help of the temperature sensitive plasmid pKC1139 [29]. The *secD* and *secF* genes were knocked out together. About 1 kb of the upstream sequence of *secD* gene and the downstream sequence of *secF* gene were cloned and linked into pKC1139 to construct the *secD-F* gene disruption plasmid pKC1139a. About 1 kb of sequences both upstream and downstream of *secDF* gene were cloned and linked into pKC1139 to construct the *secDF* gene disruption plasmid pKC1139b. Disruption plasmids pKC1139a and pKC1139b were conjugated into wild-type strain M145 through *E. coli* ET12567/pUZ8002, to knock out *secD-F* and *secDF*, respectively (Figure 1A). Plasmid pKC1139a was also introduced into ZJUZ2 (*secDF* null mutant) to construct the double mutant ZJUZ3, likewise plasmid pKC1139b was introduced into ZJUZ1 to create ZJUZ3. The gene disruption achieved in each mutant stain was verified by PCR and Southern blot analysis [30].

The 300 bp promoter regions of *secD-F* and *secDF* were cloned from genomic DNA of M145, and inserted into the promoter detecting vector pIJ8630 [31] to generate the plasmid pZJ9 and pZJ10, respectively. The plasmids were introduced into M145 to detect the activity of the *secD-F* and *secDF* promoters. The plasmid pIJ8630 was used as negative control and the plasmid pLM1 with promoter ermEp* was used as positive control [32].

The expression plasmid pZJ2 was reconstructed from pIJ8668 [31] and pHZ1272 [33], with *rep* ^pIJ101^ and *rep* ^pUC^ replicon, *oriT* fragments and antibiotic resistance genes *tsr* and *aac3(IV)*. The *xlnA* gene (SCO5931) was amplified from genomic DNA of M145 with its own promoter and His_6_ tag in C terminal, and was then inserted into pZJ2 to create the *xlnA* expression plasmid pZJ6. Plasmid pZJ6 was introduced into the wild type strain M145, and into the *secD-F* and/or *secDF* disrupted strains ZJUZ1, ZJUZ2 and ZJUZ3. The *amlC* gene (SCO7019) was amplified from genomic DNA of M145, then inserted into pLM1 and replaced the *egfp* gene, resulting in the *amlC* expression plasmid pZJ7. Plasmid pZJ7 was introduced into the wild type strain M145, and into the *secD-F* and/or *secDF* disrupted strains ZJUZ1, ZJUZ2 and ZJUZ3.

### Protein analysis and enzyme assay

For detecting the promoter activity, *S. coelicolor* mycelia were collected after incubation of spores in liquid TSB medium or on cellophane overlaid on R2YE medium. The collected mycelia were resuspended in lysis buffer (100 mM NaH_2_PO_4_, 10 mM Tris, pH 8.0, 1 mM phenylmethylsulfonyl fluoride), then lysed by ultrasonication [32]. Total protein concentration was determined using Bradford Protein Assay Kit (Invitrogen). About 20 µg total protein was separated by SDS-PAGE for Coomassie Blue staining and Western blot analysis [30] with anti-green fluorescent protein (α-GFP) antibody (Proteintech Group).

For assay of xylanase activity, *S. coelicolor* strains were grown in liquid M14 medium. Xylanase activity in supernatants was measured by the dinitrosalicylic acid method. One unit (IU) of enzyme activity is defined as the amount of enzyme required to release 1 mmol of reducing sugars (D-xylose) in 1 min at 57°C [27]. XlnA protein in supernatants was also analyzed by SDS-PAGE for Coomassie Blue staining and Western blot analysis [30] with anti-His antibody (Tiangen).

For semi-quantitatively assay of extracellular amylase activity, 20 µl spores (1×10^10^ cfu/ml) of *S. coelicolor* strains were cultured on solid MM medium (containing 0.2% soluble starch as the carbon source) at 30°C. The inoculated plates were grown for 120 hours, then they were stained with Lugol’s solution (5% I_2_ and 10% KI mixed in distilled water) for 30 min. The diameters of the transparent zones were measured to determine the relative activity [34], [35]. All measurements were depicted as a percentage of the extracellular amylase activity of ZJUZ27, which contains the *amlC* expression plasmid pZJ7 in the wild-type *S. coelicolor* strain. Each data point was measured by three times.

### RNA isolation, RT-PCR and quantitative RT-PCR

The total RNA of *S. coelicolor* was isolated from the mycelia grown in YEME medium. RNA was prepared with RNAprep pure Cell/Bacteria Kit (Tiangen) according to the manufacturer’s instructions. The genomic DNA was removed by RNase-free DNase I (Takara), and the RNA concentration was determined by measuring the OD_260_/OD_280_ in a spectrophotometer.

RT-PCR was performed in two steps: cDNA was made from 2 µg total RNA using M-MLV Reverse Transcriptase (Takara) according to the manufacturer’s manual. Then cDNA was amplified by PCR with rTaq DNA Polymerase (Takara). Amplification and detection by quantitative RT-PCR (qPCR) were performed on an Eppendorf realplex 2 Mastercycler machine (Eppendorf), and the synthesized cDNA from the first step was amplified with SYBR Premix Ex Taq (Takara) according to the manufacturer’s instructions. The principal sigma factor coding gene (*hrdB*), which is a housekeeping gene in *S. coelicolor*, was used as the internal control: all values were normalized as the relative transcript level to *hrdB*. All reactions were performed in triplicate.

### Bioinformatic analysis

The putative SecD, SecF and SecDF protein sequences of *S*. *coelicolor* were retrieved from StrepDB (http://strepdb.streptomyces.org.uk), and were used as query sequences to detect SecDF homologs in the microbial proteins of non-redundant protein database (nr) from NCBI website [36], and the Integrated Microbial Genomes (IMG) database [37] by BLASTP searches (e-value cut-off = 10^−10^, coverage = 70%). The SecD and SecF homologs of other taxa were obtained from clusters of orthologous groups (COG) family COG0341 and COG0342, respectively [38]. Protein alignment was performed using ClustalW with default parameters [39]. Protein domain predictions were made using Pfam [40] and SMART analysis [41] (Simple Modular Architecture Research Tool; http://smart.embl-heidelberg.de/). Prediction of transmembrane helices in proteins was performed using TMHMM [42]. For promoter analysis of *secDF* and *secD-F* genes, the sequences 300 bp upstream of the coding regions were analyzed using the regulatory sequence analysis tools website [43] (http://www.fruitfly.org/seq_tools/promoter.html).

### Evolutionary analysis

The amino acid sequences of SecD and SecF were concatenated into single sequence SecD-F. Full-length sequences were aligned with ClustalW [39] with default settings. Then, poorly aligned regions were removed using Gblocks v0.91b [44] with the following settings: maximum number of contiguous non-conserved positions allowed = 8; minimum length of a block allowed = 5. The neighbor-joining (NJ) tree was constructed from the alignment using the MEGA version 6.0 [45] with the parameters: p-distance model for protein sequences and pairwise deletion option for gaps. A maximum likelihood (ML) tree was built from the alignment by PhyML version 3.0, which was available at PhyML webservice (http://www.phylogeny.fr) [46]. Branch supports for phylogenetic trees were determined using 1000 bootstrap replicates. The model archaeal strain (*Haloferax volcanii*) was used as outgroup.

To identify genes neighboring the two SecDF types, genomic synteny among 14 *Streptomyces* species with complete genome sequences was investigated using the gene neighborhood tool in the Integrated Microbial Genomes system provided by the US Department of Energy Joint Genome Institute [37] (http://img.jgi.doe.gov/cgi-bin/m/main.cgi).

## Results and Discussion

### Two forms of SecDF homologs occur in *S. coelicolor*

**Figure 2:**
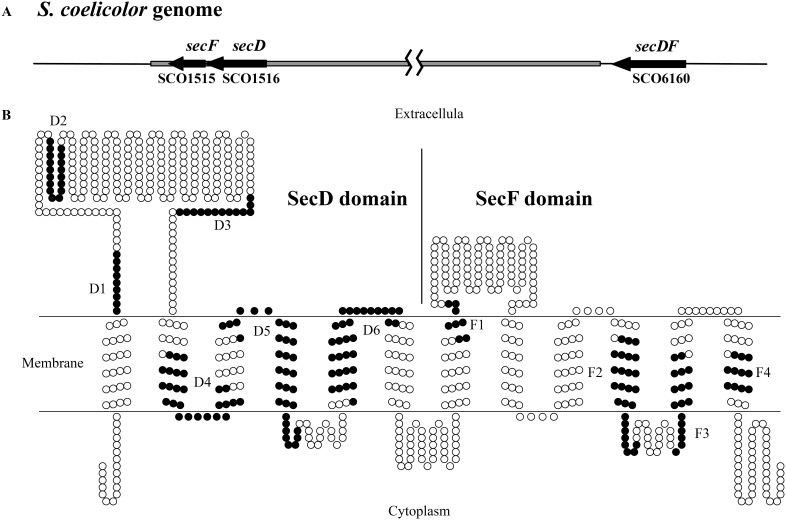
The gene location and predicted topology of *S. coelicolor* SecDF homologs. (A) Schematic diagrams of the location of *secDF* homologous genes in *S. coelicolor* genome. The core region of *S. coelicolor* is indicated in gray box. (B) The predicted topology of *S. coelicolor* SecDF protein. The conserved domains D1–D6 and F1–F4, which are presented in all known SecD and SecF proteins, are highlighted in black color.

Analysis of the complete genome sequence of *S. coelicolor* [26] identified three genes closely related to *secD* (SCO1516), *secF* (SCO1515) and *secDF* (SCO6160), with GC content 69.6%, 67.4% and 73.0%, respectively (Figure 2A). The location of *secD* and *secF* ORFs (2,838 nucleotides in length) are adjacent to each other in the conserved chromosome core region. The two ORFs are separated by an intergenic sequence of 3 nucleotides (GAG). In contrast, *secDF* is located in the variable terminal region. The *secD* gene encodes a protein of 570 amino acids, which is similar to *E. coli* SecD (26% identity). The *secF* gene encodes a protein of 373 amino acids, which is similar to *E. coli* SecF (31% identity). *S. coelicolor secDF* encodes a predicted membrane protein of 795 amino acids, which shows 30% identity to the SecDF protein of *B. subtilis*. Moreover, analysis of 17 *Streptomyces* species with complete genome sequences revealed that SecD and SecF are presented in all these species, but SecDF is absent from seven of these species (Table S2 in File S1). These results suggest that SecD/SecF homologs might play a more important role than SecDF in *Streptomyces* and undergo stabilizing selection.

The SecD, SecF and SecDF homologs of *S. coelicolor* were analyzed using TMHMM [42] to predict their membrane topologies. Consistent with the experimentally derived topologies of *E. coli* SecD and SecF, the *S. coelicolor* SecD and SecF were predicted to encode integral membrane proteins with six transmembrane segments. The fused SecDF is predicted to contain 12 transmembrane helices and two large extracellular domains (Figure 2B). The amino and carboxyl termini of these proteins are predicted to be located in the cytoplasm, and substantial extracellular domains are present between transmembrane helices 1 and 2 in all of them, besides 7 and 8 in SecDF. The first large size periplasmic loop has been shown to be involved in facilitating the formation of stable, folded conformations in the translocated Sec substrates [47].

### Transcriptional analysis of the *secDF* homologous genes

For comparative analysis of *secD*, *secF* and *secDF* transcription, total RNA was isolated from *S. coelicolor* M145 after 36 h cultivation in YEME medium. The transcript abundance for *secD-F* and *secDF* genes was measured by RT-PCR and calibrated with *hrdB* gene (Figure 1B). Both *secD* and *secF* were highly transcribed in the wild type strain M145, whereas the transcripts of *secDF* were not detectable after the same 30 cycles of RT-PCR. This result indicated the level of *secDF* transcripts was too low to be detected by RT-PCR. However, when the PCR cycle number was increased to 35, the *secDF* transcript became detectable, suggesting that *secDF* gene was barely expressed in the wild-type strain. Overall, in the wild-type strain, the transcript levels of *secD* and *secF* are much higher than those of *secDF*.

Furthermore, promoter prediction for *secD-F* gene identified a putative promoter sequence (tcggggcgtgacgacccgctcacgcggggcacattcgccggagcaggaac) that starts from 115 bp upstream of the *secD* start codon, but no putative promoter sequence was found for the *secDF* gene. To validate the promoter activity, we cloned a 300 bp fragment of the DNA upstream of the *secD-F* and *secDF* start codons (ATG), and inserted them in the upstream of *egfp* gene on the *Streptomyces* promoter-probe plasmid pIJ8630 [31], yielding plasmids pZJ9 and pZJ10, respectively. The plasmids were introduced into M145 to create strains ZJUZ40 and ZJUZ41. The strains LM6 [32], carrying a plasmid with the constitutive promoter ermEp* [48], and ZJU39, carrying a plasmid with no promoter upstream of *egfp* gene, were used as positive and negative controls, respectively. Comparison of the transcription levels by qPCR revealed that the promoter activity of *secD-F* was about 60% of the strong promoter activity of ermEp*, whereas the promoter activity of *secDF* was barely detectable (Figure S1A in File S1). Western blot analysis was performed to analyze the expression of EGFP protein after 24, 36, 48 and 72 hours of growth (Figure S1B in File S1). The expression of EGFP in ZJUZ40 was nearly constant over time, but EGFP was barely detected by western blot in ZJUZ41. Therefore, the distinct expression pattern of *secDF* and *secD-F* was reflected by their promoter activities. The different promoter activities of *secDF* and *secD-F* indicated different transcription levels between them, suggesting *secDF* and *secD-F* might have different functions in the protein translocation process.

**Figure 3:**
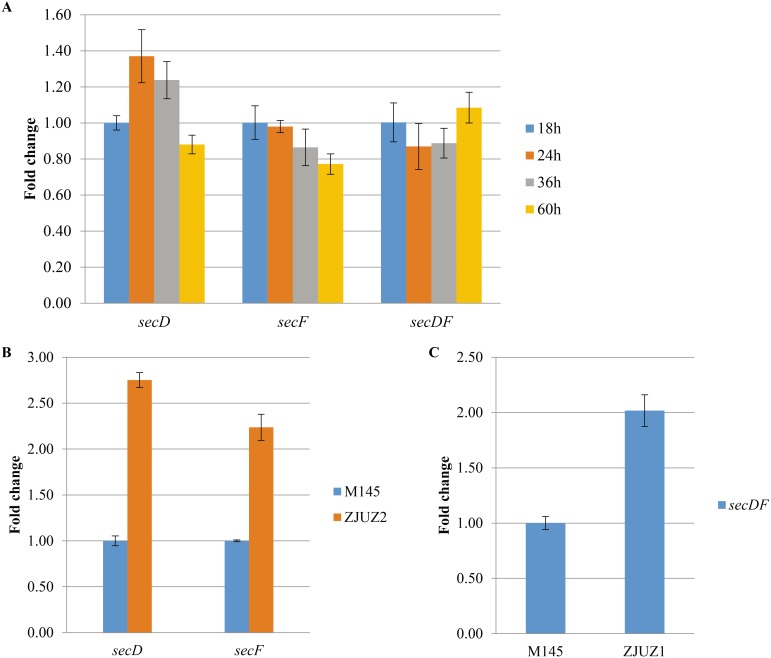
qPCR analysis of gene expression profiles of *secD*, *secF* and *secDF* in *S. coelicolor* wild type and mutant strains. (A) qPCR analysis of gene expression profile of *secD*, *secF* and *secDF* in *S. coelicolor* M145 alone the time of cultivation. (B) qPCR analysis of *secD* and *secF* gene expression in M145 and ZJUZ2. (C) qPCR analysis of *secDF* gene expression in M145 and ZJUZ1. Gene *hrdB* was used as a reference gene.

To evaluate whether the transcription of *secDF* genes depends on the growth phase, we carried out time-course expression analyses of *S. coelicolor* wild type strain M145. The strain was cultivated in YEME medium, and total RNA was isolated after 18, 24, 36 and 60 hours (Figure 3A). qPCR revealed that *secD*, *secF* and *secDF* were transcribed throughout the time of cultivation with nearly constant transcription levels, suggesting that these genes are expressed constitutively. The constant expression levels of these auxiliary components of Sec systems indicated their important roles in different growth phases of *Streptomyces*.

### Disruption of *SecDF* homologous genes and their functional effects

It has been shown that while mutations in the *E. coli secDF-yajC* operon result in a general protein secretion defect, this operon is not essential for viability [18], [25]. To determine the function of two SecDF homologs in *S. coelicolor*, we deleted each *secDF* homologous gene, creating three knock-out strains ZJUZ1, ZJUZ2 and ZJUZ3 with *secD-F*, *secDF*, and both *secD-F* and *secDF* deleted respectively (Figure 1A). These mutants were constructed by homologous recombination approach (see Materials and Methods) and were verified by PCR and Southern blot (Figure S2 in File S1).

The growth of obtained mutants with *secD-F* and/or *secDF* genes disrupted were not impaired when inoculating on four culture media, MM, SMMS, MSF, and R5, which demonstrated that the *secDF* homologous genes were not essential for the growth of *S. coelicolor* under the conditions tested (Figure S3 in File S1). As both *E. coli* strains with disrupted *secD* and/or *secF* genes, and *B. subtilis* strains with a disrupted *secDF* gene are cold-sensitive for growth and barely viable at 37°C [23], [49], [50], we tested the growth of the *S. coelicolor* mutants with disrupted *secDF* homologous genes at several temperatures (23°C, 30°C, 37°C). However, the mutants did not show reduced growth rates compared with the wild type strain at each temperature (data not shown). The fact that the growth of the *S. coelicolor* mutant with *secD-F* and *secDF* genes disrupted together is not severely impaired indicates that neither SecD-F nor SecDF is required for translocation of the essential proteins for viability and growth in these surveyed conditions.

### Deletion of the *S. coelicolor secDF* homologous genes results in a Sec-specific protein export defect

**Figure 4:**
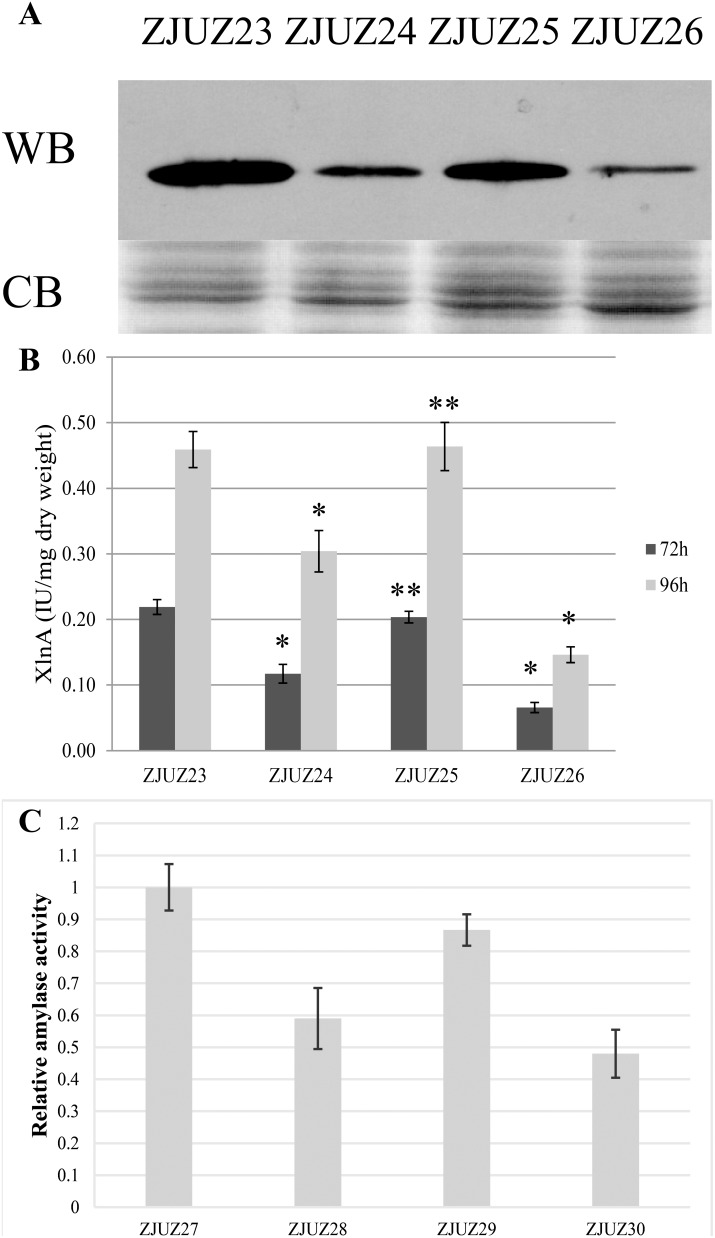
Assay of extracellular XlnA and AmlC activity and protein amounts. (A) Detecting XlnA protein secretion by SDS-PAGE and Western blot. WB: Western blot; CB: Commassie Blue Staining. ZJUZ23-26 strains were cultivated for 96 h. (B) Assay of extracellular XlnA activity. ZJUZ23-26 strains were cultivated for 72 h and 96 h. *: p<0.05, **: p>0.05, Chi-squared test. (C) Relative amylase activity from ZJUZ27, ZJUZ28, ZJUZ29 and ZJUZ30. The diameters of the transparent zones around colonies were measured to determine the relative activity.

In order to test the effects of the SecDF (Δ*secDF*) or SecD-F (Δ*secD-F*) absence on protein translocation in *S. coelicolor* mutants, we tested transport efficiency using the Sec-dependent substrate Xylanase A (XlnA) and Amylase C (AmlC) as a reporter protein. XlnA is secreted by the Sec-dependent pathway in *Streptomyces* to hydrolyze the β-1,4-D-glycosidic bonds of xylan in plant cell walls [51]. A vector, designated pZJ6, was constructed to express XlnA with His_6_ tag on its C terminus. The vector pZJ6 was transformed into *S. coelicolor* M145, ZJUZ1, ZJUZ2 and ZJUZ3, and the transformants were designated ZJUZ23, ZJUZ24, ZJUZ25, and ZJUZ26, respectively. When this construct was expressed in the four strains after cultivation for 96 hours, the culture supernatants have comparable protein profiles and similar amounts of proteins, as detected by SDS-PAGE, as shown in the lower part of Figure 4A. As SecDF was generally supposed to affect protein translocation efficiency, which can only be detected by pulse-chase method, but not by SDS-PAGE. Therefore, there is no significant difference detected between mutant and wild-type strains in this study.

Western blot analysis was performed to detect the secretion level of XlnA-His_6_ in the supernatant of *S. coelicolor* cultures (upper part of Figure 4A). As it was expected, XlnA was found to be efficiently translocated into the culture supernatant of *S. coelicolor* strain ZJUZ23, which was constructed from the wild type strain M145. As compared to that of strain ZJUZ23, the *S. coelicolor* strain ZJUZ25 (Δ*secDF*) secreted levels of XlnA into the medium that were comparable to the wild-type construct. In contrast, the levels of mature protein in the culture supernatant of the strain ZJUZ24 (Δ*secD-F*) and ZJUZ26 (Δ*secD-F* & Δ*secDF*), were present at substantially reduced levels relative to the wild-type strain, suggesting a translocation defect. Moreover, the levels of protein XlnA in the supernatant of strain ZJUZ26, which lacks both *secDF* and *secD-F*, were reduced more dramatically than that in the strain ZJUZ24, which lacks only *secD-F*. This suggests that SecDF plays an important role in promoting protein translocation when the *secD-F* was depleted.

To confirm these results, the XlnA activity in supernatant of each strain was measured at two time points (72 and 96 hours, respectively) (Figure 4B). There is no significant difference in the amount of secreted xylanase between strain ZJU25 with *secDF* deleted and strain ZJU23 (*P*>0.05, Chi-squared test). However, the strain ZJUZ24 lacking SecD-F has a significantly lower XlnA activity as compared to strain ZJUZ23 (*P*<0.05, Chi-squared test). The amount of secreted xylanase was reduced to 54% (72 h) and 66% (96 h), respectively. As expected, the XlnA activity was dramatically reduced to about 0.1 U/mg dry weight (about 30% of ZJUZ23) in strain ZJU26 with both *secD-F* and *secDF* deleted. As *S. coelicolor* contains at least three xylanases (XlnA, XlnB and XlnC), the XlnA and XlnB are secreted by Sec pathway, whereas the XlnC is secreted by TAT pathway [52]. The basal enzyme activity of total secreted xylanase in *S. coelicolor* is about 0.1 U/mg dry weight (data not shown). As the reduced amounts of extracellular xylanase activity in strain ZJUZ26 are similar to this basal value, it seems probable that disruption of both *secD-F* and *secDF* genes almost completely inhibited XlnA protein secretion.

We further tested the secretion of AmlC that belongs to the 1,4-a-D-glucan glucanohydrolase family in *S. coelicolor* [53]. The α-amylase was also secreted by Sec pathway in bacteria [54], [55]. A vector, designated pZJ7, was constructed to overexpress AmlC protein. The vector pZJ7 was transformed into *S. coelicolor* M145, ZJUZ1, ZJUZ2 and ZJUZ3, and the transformants were designated ZJUZ27, ZJUZ28, ZJUZ29, and ZJUZ30, respectively. The extracellular amylase activity was detected by Lugol’s solution stain in a semi-quantitative manner (Figure S4 in File S1) [34]. The relative amylase activity was also decreased in *secD-F* and/or *secDF* mutants compared to the wild-type strain, and showed the similar pattern to that of XlnA (Figure 4C). Therefore, the *secD-F* and/or *secDF* mutants caused the secretion defect of XlnA and AmlC, two Sec-specific proteins.

The SecDF complex is the accessory component of the Sec translocation system. Generally, SecDF plays important role in protein export by affecting protein translocation efficiency [23], [25]. The two forms SecDF homologs in *S. coelicolor* are required for efficient secretion of XlnA and AmlC. Since the two SecDF homologs in *S. coelicolor* seem to be redundant, we examined whether disruption of *secDF* homologous genes affects the transcription level of each other. qPCR analyses showed the transcription level of *secD*/*secF* in ZJU2 (Δ*secDF*) and *secDF* in ZJU1 (Δ*secD-F*) was relatively higher than that in the wild-type strain M145 (Figure 3B, C). Taken together, these results suggest that there is some degree of functional redundancy between the two SecDF homologs. However, SecD-F has a much more prominent role than SecDF in the Sec-dependent protein export and the complementation of two forms of SecDF homologs may contribute to higher secretion efficiency of *S. coelicolor*.

### Evolution and origin of *SecDF* homologous genes in *S. coelicolor*

**Figure 5:**
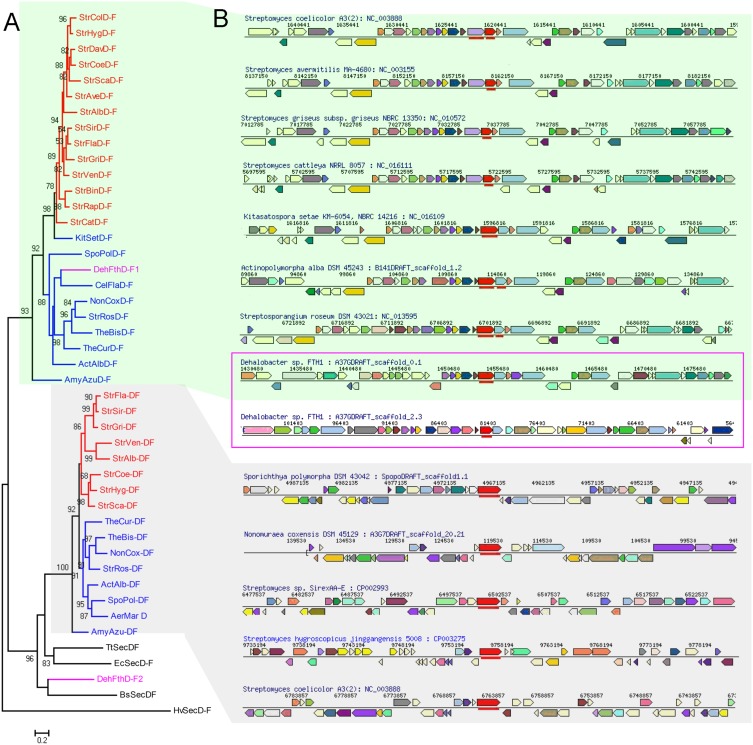
Phylogeny and gene synteny of *Streptomyces* SecDF homologs. (A) Phylogenetic tree based on the amino acid sequences of SecDF homologues identified by BLAST searches (see Methods) from those species with whole genome sequences available. Background colors refer to two different clades of SecDF and SecD-F. Bootsrtap support values greater than 50% are depicted in tree branches. Streptomyces branches are shown red, Actinobacteria branches are blue and *Dehalobacter* branches are pink. Species names are abbreviated as given in Table S2 in File S1. (B) Chromosome regions encompassing secDF gene are depicted by black lines for some species in the phylogenetic tree. The *secD* or *secDF* homologous genes are shown as red arrow in the center, and the orthologous genes between these species are depicted in the same color according to IMG database (see Methods).

Previous studies suggested that membrane proteins, including SecD and SecF, might have evolved through gene duplication and fusion events [21], [56]. To investigate the origin of two copies of *secDF* homologous genes in *S. coelicolor*, we performed BLASTp searches for SecD, SecF and SecDF homologs in various organisms (see Methods). The BLAST results were sampled to include representatives from major bacterial lineages, especially those species whose genomes have been completely sequenced. Maximum likelihood (ML) and neighbor-joining (NJ) phylogenetic trees were reconstructed for the *S. coelicolor* SecDF homologs, and both trees were congruent in topology (Figure 5A). The trees for the SecDF homologs from various taxa showed that a cluster of proteins were grouped into two well-supported distinct clades, SecDF and SecD-F (Figure 5A). The group SecDF is mainly composed of the fusion protein SecDF, while the group SecD-F is comprised exclusively of separated proteins SecD and SecF. Notably, both clades were comprised of those representative species from the phylum Actinobacteria, except *Dehalobacter sp.* FTH1 which belongs to the phylum Firmicutes. Therefore, these two forms of SecDF homologs in *S. coelicolor* seem to have evolved early in or before the divergence of extant Actinobacteria species.

Observation of the gene order within the genomic vicinity of *secD-F* in several *Streptomyces* genomes reveals similar neighboring gene identities and orders for both *secD-F* homologous types (Figure 5B). In addition, the genes surrounding *secD-F* in all species of the clade SecD-F show a high degree of synteny. In contrast, the genomic neighborhood of *secDF* gene in *S. coelicolor* does not show synteny with that of the fused type from other closely related taxa. Our previous studies have shown that a core region of genome has been extraordinarily conserved and undergone strong purifying selection to maintain gene contents in *Streptomyces* [1], [2]. As the *secD* and *secF* genes are located in the core region, they tended to be conserved in *Streptomyces* genomes. In contrast, the *secDF* genes in many *Streptomyces* species are located at terminal plastic regions of genomes, and they tend to be acquired or lost by chromosomal rearrangement events. In addition, the phylogenetic analysis shows that the fused SecDF from the phylum Actinobacteria were more closely related to those homologs from other bacteria (*E. coli*, *B. subtilis* and *Dehalobacter sp.*) than to the separated SecD-F from same phylum (Figure 5). Taken together, these results indicated that *secD-F* may have arisen through the divergence and vertical transmission of existing components from an ancestral species of *Streptomyces*, while *secDF* may have been introduced into the genome of an ancestral species of Actinobacteria by horizontal gene transfer (HGT). The acquisition of a second *secDF* copy may render a functional benefit to the organism by enhancing protein transport capacity. However, if the *secDF* gene performs a redundant function it would easily become non-essential when the environment changed. This would lead to a relaxed constraint on the *secDF* gene, and it could have been lost in some species (such as *S. avermitilis*) during the evolution of *Streptomyces*.

Interestingly, *Dehalobacter sp.* FTH1, which belongs to the phylum Firmicutes, contains two copies of separated SecD and SecF homologs, and the phylogenetic analysis shows one of them is closely related to the fused SecDF and the other is related to the separated SecD/SecF of *Streptomyces* (Figure 5). The alternative scenario is that the *secD* and *secF* genes in the common ancestor of Firmicutes and/or Actinobacteria may have undergone additional gene duplication, and one pair of the duplicate genes were further fused in the Actinobacteria ancestor, leading to the two forms of *secDF* homologous genes in *S. coelicolor*.

**Figure 6:**
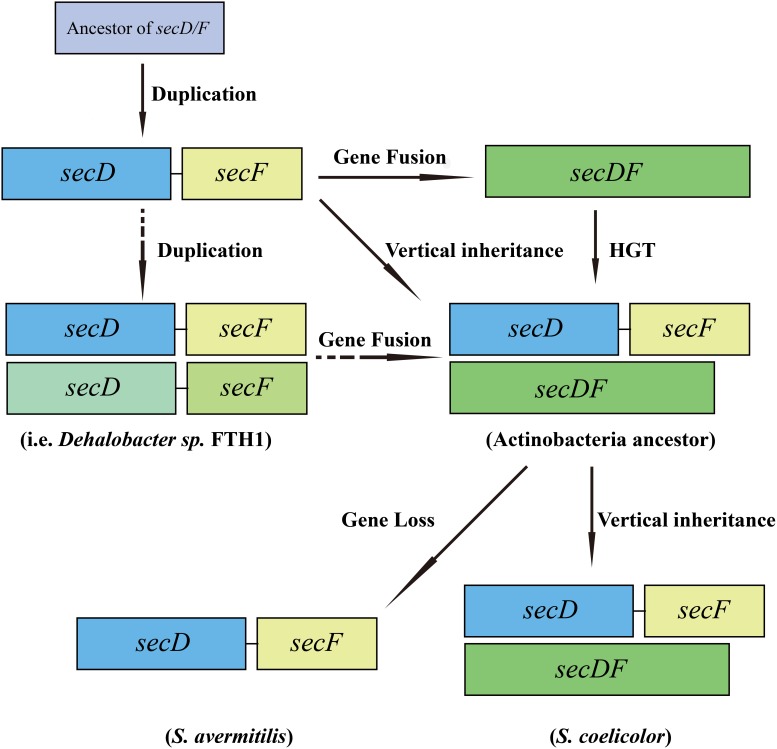
Proposed schematic model of *secDF* homologs evolution in *Streptomyces*.

Taken into consideration of earlier studies [20], [56], a potential evolutionary history of the two forms of *secDF* homologs in *Streptomyces* species could be hypothesized as follows (Figure 6). A single primitive gene (ancestor of *secD/F*) existed first. This gene was then duplicated to form two homologs, *secD* and *secF* (such as in *E. coli*). In a few genomes, a fusion of these two genes into *secDF* occurred later (such as in *B. subtilis*). The Actinobacteria ancestor inherited the separated form of *secD* and *secF* genes via vertical transmission, but acquired an additional copy of fused *secDF* gene by HGT. In the later evolutionary process, the fused *secDF* gene may have become redundant and been lost in some *Streptomyces* species. Alternatively, it is also plausible that both the fused and separated forms existing in *Streptomyces* may have been derived from the sequential gene duplication and fusion events occurring in an early progenitor of Actinobacteria. However, this seems unlikely as most extant bacteria have only one form of *secDF* homologous genes, either in separated or fused form. Furthermore, the separated and fused *secDF* genes, regardless of their origin, have diverged from each other, as reflected in their striking sequence differences. Clearly, more work is required to confirm this evolution hypothesis and to determine whether other functionalities are influenced by the presence or absence of SecDF proteins.

## Supporting Information

File S1 **Figures S1–S4, Tables S1 and S2.** Figure S1. Identify the activities of secD-F and secDF gene promoters and gene expression profile. (A) Expression level of egfp in LM6 and ZJUZ39-41 by qPCR, the expression of egfp in LM1 was set as reference. (B) Detecting the expression of EGFP protein alone the time of cultivation by SDS-PAGE and Western blotting. WB: Western blotting; CB: Commassie Blue Staining. Figure S2. PCR and Southern blot verification of gene knock-out. (A) PCR analysis for disruption of *secD-F* genes. The primer pair *secD*_p_F vs *secF*_right_arm_R (Table S1) was used. (B) PCR analysis for disruption of *secDF* gene. The primer pair *secDF*_p_F vs *secDF*_right_arm_R (Table S1) was used. (C) Southern blot analysis for disruption of *secD-F* genes, digested by *Bam*HI. (D) Southern blot analysis for disruption of *secDF* gene, digested by *Bam*HI. Figure S3. Phenotypic analysis on morphogenesis between *S. coelicolor* wild type and mutants. (A) MM 60 h, (B) SMMS 84 h, (C) MSF 72 h, (D) R5 36 h. Figure S4. Semi-quantitative assay of extracellular AmlC activity. Strains ZJUZ27-30 were grown on MM media containing 0.2% soluble starch for 5 days before staining with Lugol’s solution. Table S1. Oligonucleotides used in this study. Table S2. Distribution of SecDF homologs in *Streptomyces* species and other species depicted in Figure 4.
(PDF)
