# Supplementary material for: Function and Evolution of Two Forms of SecDF Homologs in Streptomyces coelicolor
Source: PLoS One. 2014 Aug 20;9(8):e105237. doi: 10.1371/journal.pone.0105237 (PMC4139356; doi:10.1371/journal.pone.0105237)
Supplement: File S1 — Figures S1–S4, Tables S1 and S2. Figure S1. Identify the activities of secD-F and secDF gene promoters and gene expression profile. (A) Expression level of egfp in LM6 and ZJUZ39-41 by qPCR, the expression of egfp in LM1 was set as reference. (B) Detecting the expression of EGFP protein alone the time of cultivation by SDS-PAGE and Western blotting. WB: Western blotting; CB: Commassie Blue Staining. Figure S2. PCR and Southern blot verification of gene knock-out. (A) PCR analysis for disruption of secD-F genes. The primer pair secD_p_F vs secF_right_arm_R (Table S1) was used. (B) PCR analysis for disruption of secDF gene. The primer pair secDF_p_F vs secDF_right_arm_R (Table S1) was used. (C) Southern blot analysis for disruption of secD-F genes, digested by BamHI. (D) Southern blot analysis for disruption of secDF gene, digested by BamHI. Figure S3. Phenotypic analysis on morphogenesis between S. coelicolor wild type and mutants. (A) MM 60 h, (B) SMMS 84 h, (C) MSF 72 h, (D) R5 36 h. Figure S4. Semi-quantitative assay of extracellular AmlC activity. Strains ZJUZ27-30 were grown on MM media containing 0.2% soluble starch for 5 days before staining with Lugol’s solution. Table S1. Oligonucleotides used in this study. Table S2. Distribution of SecDF homologs in Streptomyces species and other species depicted in Figure 4. (PDF) [file pone.0105237.s001.pdf]

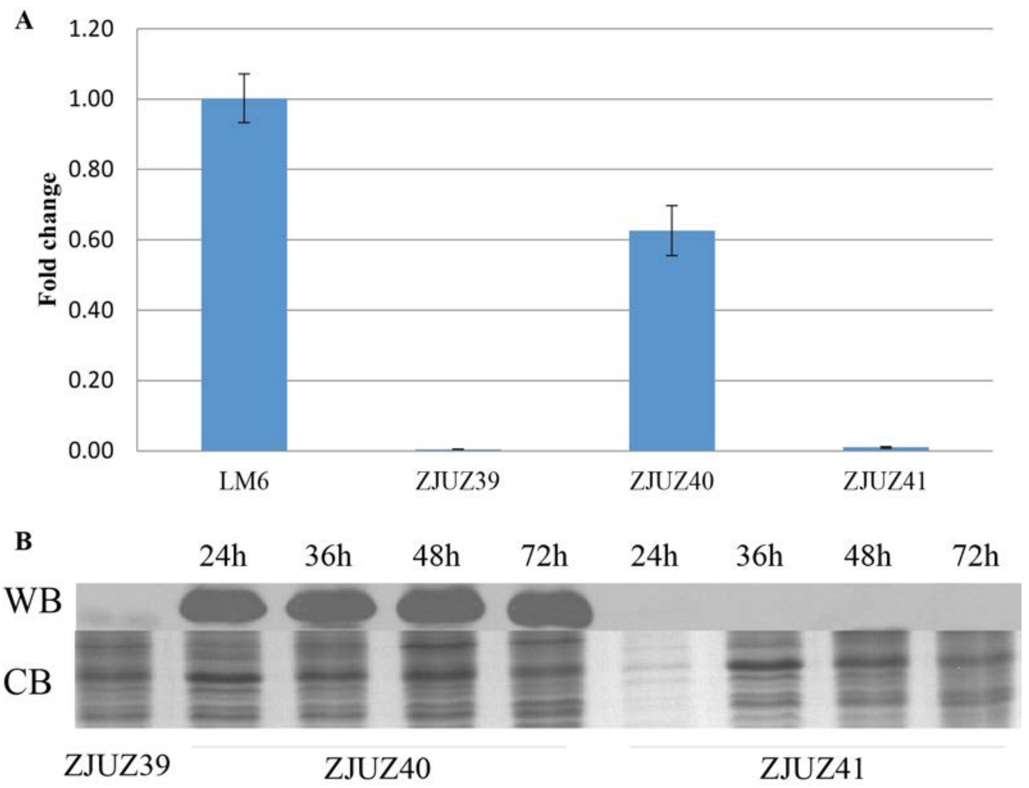

Figure S1. Identify the activities of *secD-F* and *secDF* gene promoters and gene expression profile. (A) Expression level of *egfp* in LM6 and ZJUZ39-41 by qPCR, the expression of *egfp* in LM1 was set as reference. (B) Detecting the expression of EGFP protein alone the time of cultivation by SDS-PAGE and Western blotting. WB: Western blotting; CB: Commassie Blue Staining.

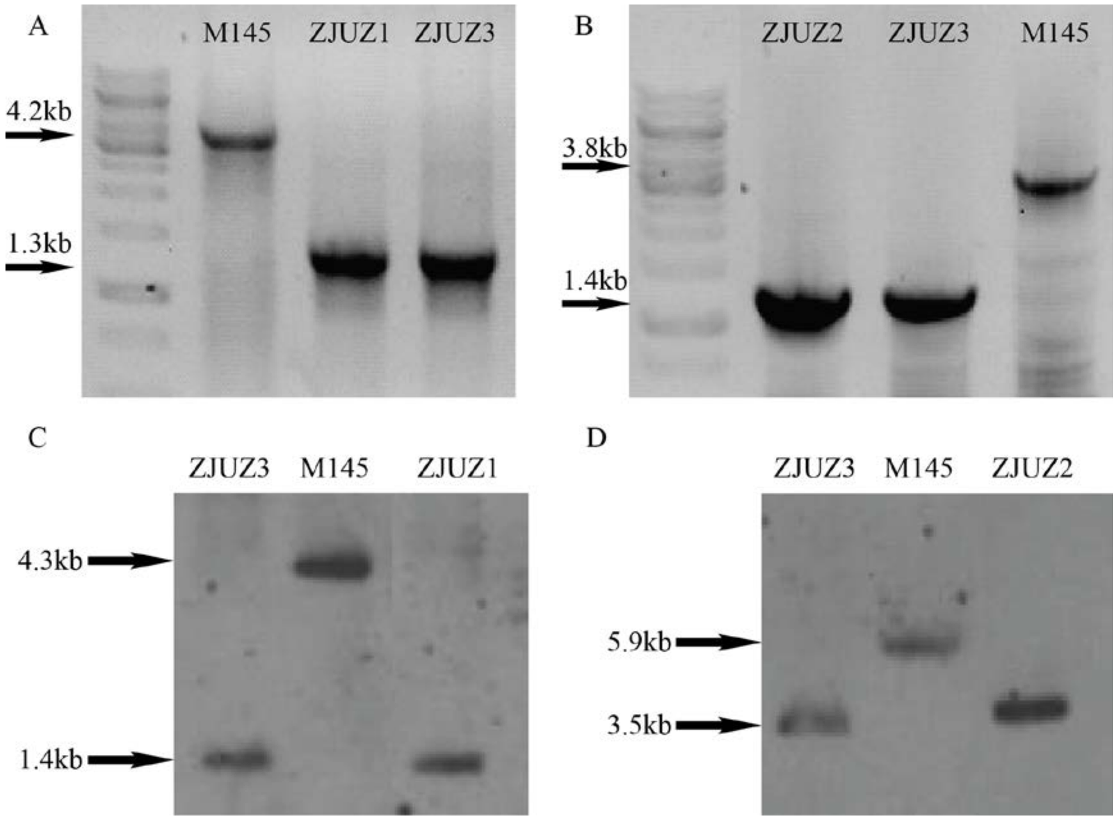

Figure S2. PCR and Southern blot verification of gene knock-out. (A) PCR analysis for disruption of *secD-F* genes. The primer pair *secD*_p_F vs *secF*_right_arm_R (Table S1) was used. (B) PCR analysis for disruption of *secDF* gene. The primer pair *secDF*_p_F vs *secDF*_right_arm_R (Table S1) was used. (C) Southern blot analysis for disruption of *secD-F* genes, digested by *Bam*HI. (D) Southern blot analysis for disruption of *secDF* gene, digested by *Bam*HI.

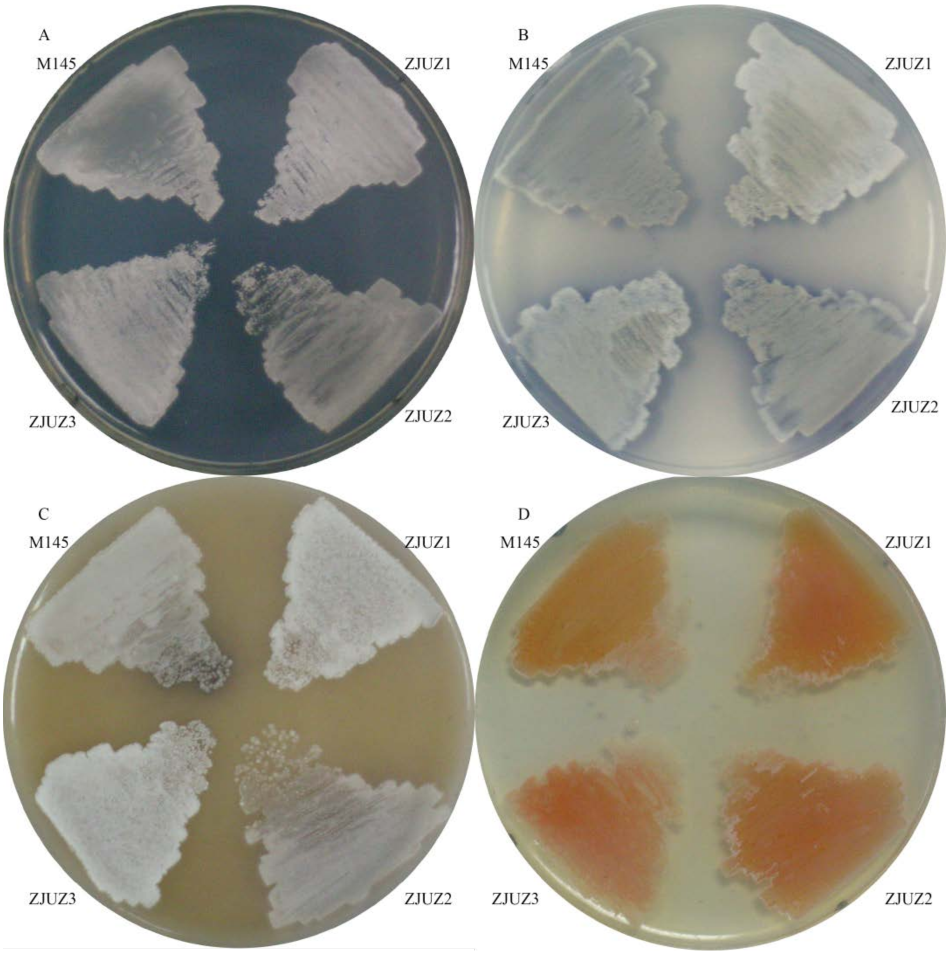

Figure S3. Phenotypic analysis on morphogenesis between *S. coelicolor* wild type and mutants. (A) MM 60h, (B) SMMS 84h, (C) MSF 72h, (D) R5 36h.

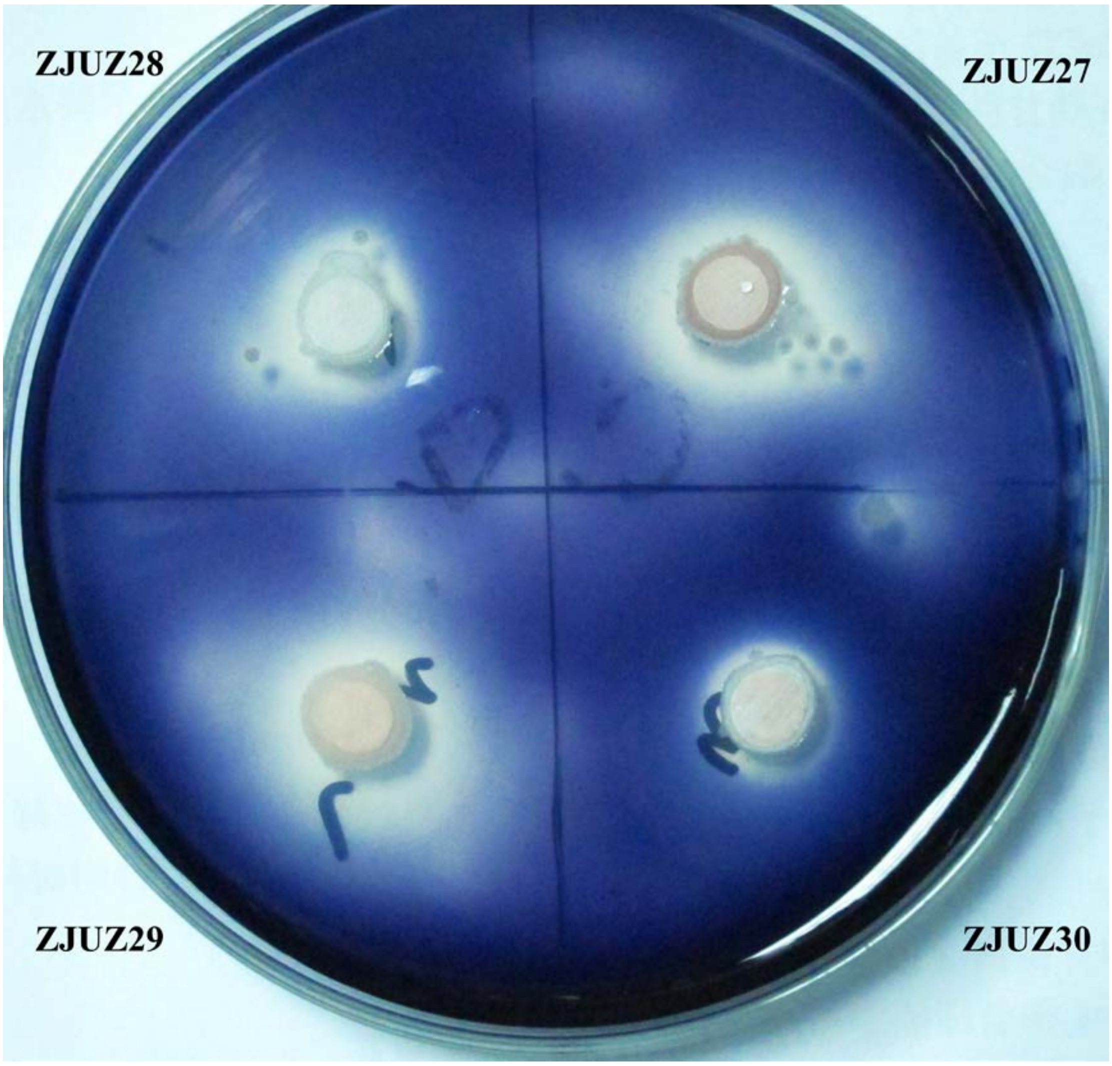

Figure S4. Semi-quantitative assay of extracellular AmlC activity. Strains ZJUZ27-30 were grown on MM media containing 0.2% soluble starch for 5 days before staining with Lugol's solution.

Table S1. Oligonucleotides used in this study.

Primer	Sequence (5'-3')	Description
*secD*_left arm_F	ACTAAGCTTCGCGTCCGCAGAGTTCAG	*secD*_left arm 5' HindIII
*secD*_left arm_R	TCATCTAGACCCGGAGGCGAACATGC	*secD*_left arm 3' XbaI
*secF*_right arm_F	TGATCTAGAGGGAAGCGCCGATGACC	*secF*_right arm 5' XbaI
*secF*_right arm_R	TGAGAATTCGACGCGGTTGGAGGAGC	*secF*_right arm 3' EcoRI
*secDF*_left arm_F	ACTAAGCTTTGCTGACGATGCACGACAAC	*secDF*_left arm 5' HindIII
*secDF*_left arm_R	TCATCTAGAGGAGCGTTTCAAGACGGGC	*secDF*_left arm 3' XbaI
*secDF*_right arm_F	TCATCTAGATAGCAGGGGCGGGCAGG	*secDF*_right arm 5' XbaI
*secDF*_right arm_R	ACTGAATTCCGGCGGACAGGGCACGG	*secDF*_right arm 3' EcoRI
*secD*_p_F	ACTTCTAGAGACGAGACGGCCGACGAGC	*secD* promoter 5' XbaI
*secD*_p_R	TTATCATATGGCCCCCGGTGAGCGCC	*secD* promoter 3' NdeI
*secDF*_p_F	TTATTCTAGACGGTCCGAGCGGTCCCCTC	*secDF* promoter 5' XbaI
*secDF*_p_R	TATTCATATGGACGGGCCTCCGGCGG	*secDF* promoter 3' NdeI
*xlnA*_F	GATGGATCCCGACTTGGGTGGGTGGTTC	*xlnA* 5' Promoter BamHI
*xlnA*_R	TCATCTAGATCAGTGGTGGTGGTGGTGGTG GGTGCGGGTCCAGCGTTG	*xlnA* 3' XbaI 6*His
*amlC*_F	TACTCTAGAATGCGATGGCTCGGACG	*amlC* 5’XbaI
*amlC*_R	TACTCTAGAGAAACCCGAACGCACCAAG	*amlC* 3’XbaI
secD-q-F	GTGTCAATGGTCTGGGTGTTTCC	qPCR primer for *secD* (148)#
secD-q-R	CGGGCTTCCTTCGAGTTCGTC	qPCR primer for *secD* (248)
secF-q-F	ACCATCACCGTGGGCATCT	qPCR primer for *secF* (529)
secF-q-R	GACCGTGTCGTAGAGCGAGTAA	qPCR primer for *secF* (630)
secDF-q-F	GCCGACTCCAACATCACCACG	qPCR primer for *secDF* (1195)
secDF-q-R	GTGACGCCGAAGCCCTTGA	qPCR primer for *secDF* (1277)
hrdB-q-F	GACCAGATTCCGGCCACTC	qPCR primer for *hrdB* (130)
hrdB-q-R	GCTCTGCGGCACTGACCAT	qPCR primer for *hrdB* (226)
egfp-q-F	CGTCCAGGAGCGCACCATCT	qPCR-*egfp* (285)
egfp-q-R	TGCGGTTCACCAGGGTGTCG	qPCR-*egfp* (376)

Numbers in the brackets indicate the position of 5’ terminal nucleotide of primers in the gene coding regions

Table S2. Distribution of SecDF homologs in *Streptomyces* species and other species depicted in Figure 4.

Strain	Abbreviation	SecD	SecF	SecDF
Streptomyces albus	StrAlb	+	+	+
Streptomyces avermitilis	StrAve	+	+	-
Streptomyces bingchenggensis	StrBin	+	+	-
Streptomyces cattleya	StrCat	+	+	-
Streptomyces coelicolor	StrCoe	+	+	+
Streptomyces collinus	StrCol	+	+	-
Streptomyces davawensis	StrDav	+	+	-
Streptomyces flavogriseus	StrFla	+	+	+
Streptomyces fulvissimus	StrFlu	+	+	+
Streptomyces griseus	StrGri	+	+	+
Streptomyces hygroscopicus	StrHyg	+	+	+
Streptomyces sp. PAMC26508	StrPAM	+	+	+
Streptomyces rapamycinicus	StrRap	+	+	-
Streptomyces scabiei	StrSca	+	+	+
Streptomyces sp. sirexAA	StrSir	+	+	+
Streptomyces venezuelae	StrVen	+	+	+
Streptomyces violaceusniger	StrVio	+	+	-
Actinopolymorpha alba	ActAlb	+	+	+
Aeromicrobium marinum	AerMar	-	-	+
Amycolatopsis azurea	AmyAzu	+	+	+
Cellulomonas flavigena	CelFla	+	+	-
Dehalobacter sp. FTH1	DehFth	2	2	-
Kitasatospora setae	KitSet	+	+	-
Nonomuraea coxensis	NonCox	+	+	+
Sporichthya polymorpha	SpoPol	+	+	+
Streptosporangium roseum	StrRos	+	+	+
Thermobispora bispora	TheBis	+	+	+
Thermomonospora curvata	TheCur	+	+	+
Thermus thermophilus	Tt	-	-	-
Escherichia coli	Ec	+	+	-
Bacillus substilis	Bs	-	-	+
Haloferax volcanii	Hv	+	+	-
